# Viral Myocarditis as a Factor Leading to the Development of Heart Failure Symptoms, Including the Role of Parvovirus B19 Infection—Systematic Review

**DOI:** 10.3390/ijms25158127

**Published:** 2024-07-25

**Authors:** Sebastian Krych, Agata Jęczmyk, Michał Jurkiewicz, Martyna Żurek, Małgorzata Jekiełek, Paweł Kowalczyk, Karol Kramkowski, Tomasz Hrapkowicz

**Affiliations:** 1 Student’s Scientific Association, Department of Cardiac, Vascular and Endovascular Surgery and Transplantology, Faculty of Medical Sciences in Zabrze, Medical University of Silesia, 40-055 Katowice, Poland; 2 Student’s Scientific Association, Department of Descriptive and Topographic Anatomy, Faculty of Medical Siences in Zabrze, Medical University of Silesia, 40-055 Katowice, Poland; aga.jeczmyk@gmail.com; 3 Student’s Scientific Association, 3rd Department of Cardiology, Faculty of Medical Sciences in Zabrze, Medical University of Silesia, 40-055 Katowice, Poland; jurkiewicz.m@yahoo.com; 4 Zbigniew Religa Student’s Scientific Association, Department of Biophysics, Faculty of Medical Sciences in Zabrze, Medical University of Silesia, 40-055 Katowice, Poland; martynazu00@gmail.com; 5 Department of Physiotherapy, Faculty of Health Sciences, Jagiellonian University Collegium Medicum, 31-008 Cracow, Poland; malgorzata.jekielek@gmail.com; 6 Department of Animal Nutrition, The Kielanowski Institute of Animal Physiology and Nutrition, Polish Academy of Sciences, Instytucka 3, 05-110 Jabłonna, Poland; 7 Department of Physical Chemistry, Medical University of Bialystok, Kilińskiego 1, 15-089 Białystok, Poland; kkramk@wp.pl; 8 Silesian Centre for Heart Diseases in Zabrze, Department of Cardiac, Vascular and Endovascular Surgery and Transplantology, Medical University of Silesia, 40-055 Katowice, Poland; thrapkowicz@sum.edu.pl

**Keywords:** virus disease, myocarditis, heart failure, parvovirus B19

## Abstract

Myocarditis (MC) is defined as an immunological inflammatory reaction with various etiologies, clinical presentations and prognoses within the myocardium. Currently, parvovirus B19 (PVB19) has become the main factor leading to this disease, replacing the previously dominant viruses A and B. In the case of chronic heart failure with subsequent dilated cardiomyopathy, approximately 67% have a viral etiology, and most of them are the result of PVB19 infection. However, the analysis showed a correlation between PVB19 infection and the risk of developing inflammatory dilated cardiomyopathy (DCMi). PVB19 is detected in 23% of patients with DCMi. Chronic infection may also contribute to progressive left ventricular failure in patients with a history of MC. The above effect suggests the active replication of PVB19 only in heart biopsies with inflammation due to MC or DCMi. Moreover, the supply of IFN-β to suppress the active transcription of PVB19 accompanied by DCMi over a period of 6 months results in the normalization of NT-proBNP and an improvement in LVEF along with NYHA performance. The small number of reports on this topic and inaccuracies resulting from constantly conducted research and ongoing changes make it impossible to clearly answer the question of whether PVB19 is a factor inducing de novo MC and DCM or only accompanies the above conditions. However, large clinical cohort studies lead to the perception of PVB19 as a viral etiological agent capable of causing de novo MC together with DCMi.

## 1. Introduction

Myocarditis (MC) is defined as an immunological inflammatory reaction of various etiologies occurring within the myocardium, occurring in 10 to 20 cases per 100,000 people [1]. It is characterized by a diverse clinical picture and prognosis: from asymptomatic forms to life-threatening conditions such as cardiogenic shock, pulmonary edema or sudden cardiac arrest. A highly non-specific clinical course in different patients increases the risk of making an incorrect diagnosis or prolonging the diagnostic procedure, which delays the patient’s treatment, reducing the chances of survival. “2021 ESC Guidelines for the diagnosis and treatment of acute and chronic heart failure” include acute chest pain, dyspnea, features of right or left ventricular failure, arrhythmias of an unknown etiology and sudden cardiac death as clinical signs suggestive of myocarditis [2]. The consequence of variously intense immune reactions is the pathological reconstruction of the heart muscle, which may lead to dilatation of the heart chambers, disturbances in the function of the right and left ventricles and symptoms of heart failure [3]. It is estimated that in over 90% of cases, viruses are the etiological factor leading to autoimmune damage to cardiomyocytes. The above percentage has increased over the last 20 years due to the introduction of modern histological, immunological and immunohistochemical techniques and, above all, molecular tests such as polymerase chain reaction (PCR) into the routine clinical diagnosis of MS, which allowed the etiology of the disease to be determined in previously idiopathic cases of myocarditis. Currently, it is believed that over 27 viruses may be responsible for myocarditis. Currently, parvovirus B19 (PVB19) has become the main factor leading to this disease, replacing the previously dominant coxsackieviruses A and B. Moreover, it is the most frequently detected virus in diagnostic PCR (approximately 33–64% of tests) [4]. This article aims to draw the attention of clinicians to reports of new viral etiological agents (PVB19) of myocarditis and dilated cardiomyopathy, which significantly affects the diagnostic and therapeutic process.

## 2. Methods

**Figure 1:**
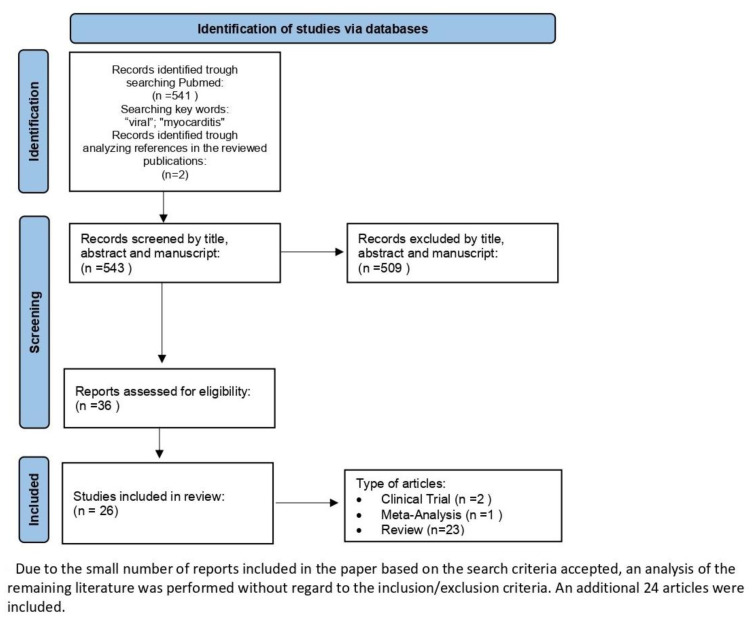
Identification of studies via databases.

Publications available on the PubMed platform were used to prepare the article. The following articles were listed: clinical trials, meta-analyses, randomized control trials, reviews and systematic reviews. The keywords used for the search were “viral” and “myocarditis”. The search range was from 2014 to 2024. Two included articles are older than the accepted scope of the search. The Hufnagel G et al. work from 2000 had significant substantive value for the ESETCID research based on a cohort of 3055 patients. The article published by Bock CT et al. from 2010 was used due to its substantive value and the author’s further contribution to expanding knowledge in the context of PVB19 infection in subsequent works analyzed in the manuscript. Taking into account the above, 541 publications were found, and 24 were used in this work. Two of them (Hufnagel G et al. and Bock CT et al.) were included by analyzing references in the reviewed publications. We included clinical trials, meta-analyses, randomized control trials, reviews and systematic reviews discussing the etiologies, pathogenesis and therapeutic management of myocarditis and cardiomyopathy, comparing PVB19 with other virus infections. Due to the lack of articles on the above topic, only clinical trials, meta-analyses and reviews were found. Articles about myocarditis or cardiomyopathy in SARS-CoV-2 infection and articles containing non-statistical analyses or with low statistical power, emphasizing the need for further research due to the inability to conclude on the basis of previous studies, were excluded. Because of the small number of reports included in the paper based on the search criteria accepted, an analysis of the remaining literature was performed without regard to the inclusion/exclusion criteria. . The keywords used for the search were “viral”, “myocarditis”, “PVB19”, “DNA”, “repair”, “heart”, “damage” and “platelets”. The search range was from 2014 to 2024. Article type criteria were not established. An additional 22 articles were included (Figure 1).

## 3. Results and Discussion

### 3.1. Viral Myocarditis

#### 3.1.1. Definition and Pathophysiology

It is an autoimmune process induced by a viral factor. The disruption of autoimmune control mechanisms results in a specific inflammatory response directed against one’s own body and viral antigens [3]. By penetrating into cardiomyocytes, viruses activate tissue inflammatory mediators via NK cells and macrophages, particularly Interleukin 1 (IL-1), Interleukin 2 (IL-2), tumor necrosis factor alpha (TNF alpha) and Interferon-gamma. Myocardial sections obtained as a result of endomyocardial biopsy show cellular infiltrates consisting of leukocytes. Depending on the type of MC, they are dominated by CD3+ T lymphocytes, monocytes or eosinophils [5]. As a result of the inflammatory process, cardiomyocytes are directly damaged by leukocytes and the substances they release, as well as microcirculation disorders caused by the activation of the endogenous coagulation system [5]. Cardiomyocyte apoptosis occurs and fibrosis occurs at the site of tissue damage [1]. In the chronic stage, it leads to the dysfunction of the heart chambers, the activation of the neurohumoral systems (sympathetic system and renin–angiotensin–aldosterone system) and the development of heart failure [1].

#### 3.1.2. Morbidity/Mortality in the Course of Viral Myocarditis

Myocarditis occurs with a frequency of 10 to 20 cases per 100,000 people [1]. The global mortality rate from myocarditis is estimated at 46,486 per 100,000 people (95% UI 39,709 to 51,824). There are no data detailing viral myocarditis, but it is known that viruses are the main etiological inflammation agent [6].

#### 3.1.3. Clinical Picture of Viral Myocarditis

Hufnagel G et al. in the ESETCID study (The European Study of Epidemiology and Treatment of Cardiac Inflammatory Diseases) draw attention to the most characteristic symptoms to help make a preliminary diagnosis of viral myocarditis, despite the highly non-specific course of this disease. These include shortness of breath (72%), stenocardial pain (32%) and cardiac arrhythmias (18%) [7,8]. MC may occur as an acute coronary syndrome, first-time symptoms of heart failure, atrioventricular conduction disturbances (clinically significant bradycardia), the exacerbation of chronic heart failure as well as cardiogenic shock or sudden cardiac death. In most cases, after the acute period and implementation of appropriate treatment, normal heart function returns and symptoms disappear. In predisposed people, the virus induces an autoimmune process leading to gradual irreversible damage to the heart muscle and dilated cardiomyopathy [2,5].

#### 3.1.4. Medical Diagnosis

The early diagnosis of MC is very important because it allows for the appropriate treatment and inhibition of the progression of the disease. MC may be suspected based on the history and physical examination, electrocardiography, laboratory tests, cardiac ultrasound and magnetic resonance imaging (CMR). The gold standard for diagnosing MC is endomyocardial biopsy, which should be performed only if there is no other way to determine the cause of myocardial disease, and its result may influence a change in treatment [9]. Biopsy is an invasive test, so it is performed quite rarely due to the possibility of complications. The non-invasive diagnostic standard in patients suspected of MC is CMR. The indication for the test is the presence of symptoms and electrocardiographic changes preceded by a viral infection, increased cardiac troponin values, no restrictive changes in coronary angiography and changes in echocardiography. According to the guidelines of the European Society of Cardiology (ESC), the diagnosis of MC requires meeting at least one clinical and one diagnostic criterion (in the absence of obstructive changes in coronary angiography and pre-existing heart disease) [2].

The diagnostic criteria include

- structural or functional changes in imaging tests (left ventricular dysfunction on cardiac ultrasound or CMR)
- the presence of edema or late gadolinium enhancement on CMR

##### Physical and Laboratory Examinations

More than 90% of patients with viral myocarditis present changes on the ECG, ST segment elevation or depression (59%), sometimes with T wave inversion or a Q wave present. In addition, approximately 10% have a bundle block [5,10]. The prolongation of the QRS time is a risk factor for death from cardiac causes [11]. “2021 ESC Guidelines for the diagnosis and treatment of acute and chronic heart failure” points out the following changes in the echocardiographic picture [2,5]:

- Structural/function abnormalities de novo
- Enlargement of the heart chambers
- Regional heart muscle contraction abnormalities
- Global left and/or right ventricle ventricular dysfunction with or without ventricular dilatation
- Myocardial oedema with wall thickness
- Pericardial effusion or thrombus formation
- Heart disease with unexplained etiology (e.g., coronary artery disease, valvular disease)

Indicators of myocardial necrosis (creatine phosphokinase and troponins) may be elevated [5].

##### Imaging Tests

The currently recommended imaging test for facilitating the diagnosis of myocarditis is cardiac magnetic resonance imaging (CMR), which requires meeting the Lake Louise Criteria (LLC) criteria included in the 2009 consensus. These include

- Myocardial edema assessed in a T2-weighted sequence. When it occurs, we observe signal enhancement in the T2-weighted sequence. Moreover, the muscle relaxation time may be prolonged in both segmental and global assessment.
- Hyperemia analyzed in a T1-weighted sequence during initial gadolinium contrast enhancement. The gain factor (SI) must be ˃4.0. T1-weighted mapping shows segmental and global extension of the relaxation time or an increase in the extracellular volume.
- Necrosis/fibrosis assessed on a T1-weighted sequence at the stage of late gadolinium contrast enhancement is marked by signal enhancement creating characteristic non-ischemic distribution patterns. Mapping in the T1-weighted sequence has features similar to those in the case of hyperemia [12].

##### Histopathological Diagnosis

It was originally based on the Dallas classification standardizing methods of tissue collection and the preparation of histopathological reports. H&E staining of the collected material is the combination of two histological stains: hematoxylin and eosin H&E. This allows CM to be classified into one of three categories:

- MC with the presence or absence of fibrosis
- Border CM
- No MC features

Due to the similarity of histopathological images of individual subtypes of myocarditis, this method is no longer preferred. Difficulties in determining the stage of inflammation, the duration of retrograde changes or the degree of cardiomyocyte loss and tissue architecture disorders are an advantage of the immunohistochemical method, which is a much more accurate technique and allows for a precise diagnosis [3].

##### Immunohistochemical Diagnosis

The immunohistochemical technique is based on the identification and marking of cells expressing selected proteins/glycoproteins on their surface. This allows for an extremely precise classification of inflammation into one of the selected subtypes:

- Acute lymphocytic myocarditis (ALM), characterized by infiltration of the myocardium by CD3-expressing T lymphocytes and associated CD68+-expressing macrophages. The tissue shows minimal signs of fibrosis. This is one of the most common subtypes.
- Chronic lymphocytic myocarditis (CLM), which is a chronic form of ALM, characterized by an intense process of tissue fibrosis with lymphocytic infiltration analogous to ALM
- Giant cell myocarditis (GCM) is an immunohistochemical subtype of inflammation associated with an autoimmune background. Myocardial infiltration by macrophages of the CD68+ subtype predominates over T lymphocytes. Additionally, leukocyte infiltration is present. This subtype is associated with a significant risk of developing dilated cardiomyopathy. The 4-year survival of patients with the above subtype without heart transplantation is 10%.
- Sarcoid myocarditis is characterized by severe infiltration of the myocardium by macrophages, leading to chronic inflammation and tissue damage.
- Eosinophilic myocarditis is a subtype of inflammation with a significant predominance of myocardial infiltration by eosinophils. It is often associated with coexisting hypereosinophilia or chronic eosinophilia due to infection. It may also be primary, as an autoimmune disease. It has a poor prognosis, similar to the giant cell form [3].

### 3.2. Cardiotropic Viruses

There are over 27 viruses directly or indirectly associated with the induction of myocarditis [4]. The most common etiological factors of MC include enteroviruses (Coxsackie A and B viruses, Echoviruses), erythroparvovirus (parvovirus B19), herpes virus (HHV6), Epstein–Barr virus (EBV), cytomegalovirus (CMV), lentivirus (HIV), hepacivirus (HCMV), influenza A and B viruses and others [13]. So far, viruses of the Coxsackie species subtype B are considered the most common etiological factor of viral myocarditis globally (10–20%) of all incidents with complications in the form of dilated cardiomyopathy [1,14]. The latest research using the PCR technique to obtain a sufficient amount of genetic material to precisely determine the type and species of the virus proves the predominant involvement of pathogens of the species parvovirus subtype B19 in the induction of viral myocarditis [4,15]. For incident cases of chronic heart failure with subsequent dilated cardiomyopathy, approximately 67% have a viral etiology, most of which are the result of PVB19 infection. The genetic material of the virus is detected in 33–64% of the PCR analyses performed [4].

#### 3.2.1. Parvovirus B19

##### Structure and Epidemiology

Parvovirus B19 is the only erythroparvovirus that has the ability to infect human cells. The genetic material consists of poorly developed single-stranded DNA (ssDNA) surrounded by an icosahedral capsid composed of 60 capsomers [16]. PVB19 is one of the most common infectious agents in childhood. PVB19-specific IgG antibodies are found in the serum of 2–15% of children under 5 years of age [17]. This value increases to 60% in adults [9]. A drastic increase in seropositivity is observed in the elderly, reaching over 90%. In children, the most common clinical picture of infection is erythema infectiosum, characterized by a facial rash and flu-like symptoms. PVB19 infection is asymptomatic in approximately 50% of children and 25% of adults This virus is associated with a significantly increased risk of serious hematological, hepatological, neurological, rheumatological, nephrological and cardiovascular complications. The prevalence of MC induced by the PVB19 was approximately 23.7% (95% CI: 18.7–29.5%), with statistical significance (OR: 4.317, 95% CI: 1.831–10.180; *p* = 0.001) [17,18].

##### Mechanism of Infection and Induction of Myocarditis

Parvovirus B19, as a vasotropic virus, does not penetrate directly into cardiomyocytes. In the cardiovascular system, the target point is the endothelial cells of myocardial blood vessels and the progenitor cells of the erythroid line [19,20]. After the symptoms of the primary infection disappear, the virus enters its latent phase. The reactivating factor is the weakening of the immune system, e.g., strep throat [20].

- NS1 and VP1/2 proteins, as well as low-molecular-weight proteins of 11 kDa and 7.5 kDa, whose function is unknown, are involved in the initiation of inflammation [9]. The NS1 protein facilitates virus replication and blocks the body’s cell cycle, leading to extensive DNA damage, ultimately resulting in apoptosis of the infected cell [9]. Additionally, the expression of NS1 protein in HEK-293 T lymphocytes stimulates the release of pro-inflammatory cytokines, IL-6 and TNF-α [19].
- VP1/2 protein with phospholipase A2 activity initiates inflammation and cell lysis [9]. The VP1 protein additionally has the ability to disrupt epithelial sodium channels. This causes an increase in endothelial ENaC activity and the swelling of endothelial tissues [21].

The NS1 helicase protein and the VP1/2 protein have the ability to induce autoimmunity processes involving T lymphocytes [9,22].

##### Pathophysiology of Myocardial Damage

The interaction of the above proteins and mechanisms (Section “Mechanism of Infection and Induction of Myocarditis”) within the endothelial cells of the coronary microcirculation vessels promotes the spread of infection in the heart muscle and the lysis of endothelial cells [9,23]. Collateral to this process, accompanied by local inflammation, cardiomyocytes are damaged. The transition of the infection from the acute to chronic phase is strictly dependent on the regenerative capacity of the endothelium and the density of the microcirculation network [9]. The degree of myocardial damage is most likely also related to the pathogen’s viremia [24]. Patients with reduced LVEF in whom viral DNA was detected in endomyocardial biopsy showed better improvement in left ventricular contractility if no virus was detected in the subsequent biopsy compared to patients with constantly detectable viral DNA in the myocardium [25]. The effect of parvovirus B19 on collateral heart failure remains unclear. In a study on a small group of 37 patients with systolic heart failure, 84% of them were positive for the presence of PVB19 [20]. PVB19 may induce fulminant myocarditis, requiring the implementation of intensive therapy, including mechanical circulatory support to compensate for cardiac performance and regenerate cardiomyocytes [26,27]. However, it should be remembered that PVB19 does not have to be the main factor involved in myocardial damage, ultimately leading to the impairment of its systolic and/or diastolic function. It is believed that PVB19 is sometimes one of the non-specific factors overlapping the background of a different inflammatory etiology [13,28,29,30]. A situation that allows for a clear diagnosis of viral myocarditis due to PVB19 infection is considered to be the detection of replication at a level of >500 copies of viral DNA in 1 microgram of DNA from myocardial tissue [13].

##### Dilated Cardiomyopathy Due to PVB19 Infection

Although PVB19 has been considered for some time as an etiological factor that may initiate or promote the development of dilated cardiomyopathy (DCM), the available literature does not contain any scientific publications regarding the risk of developing DCM due to PVB19 infection. According to reports by Maisch B et al. in patients with myocarditis, PVB19 was detected in 23% of patients with inflammatory dilated cardiomyopathy (DCMi) and in 16% of those with non-inflammatory dilated cardiomyopathy (DCM). Moreover, a significantly poorer elimination of PVB19 was observed compared to that of pathogens from the enterovirus family. Chronic infection may also contribute to progressive left ventricular failure in patients with a history of MC. In a study analyzing 1098 patients with inflammatory dilated cardiomyopathy and 2247 patients with non-inflammatory dilated cardiomyopathy, the following relationships were found (Table 1) [31].

Verdonschot J et al. showed a correlation between PVB19 infection and the risk of developing DCMi. The ability of the virus to survive in coronary endothelial cells after the acute phase of MS promotes the development of immunological inflammatory diseases that may lead to the development of DCMi. In the analyzed publications, no significant impact of DCMi induced by PVB19 on the prognosis was observed compared to the DCMi of other viral etiologies. The authors emphasize the imperfection of the research conducted so far, which makes it impossible to clearly summarize the impact of the presence and replication of the PVB19 virus on the clinical course and prognosis of patients with DCMi [32]. However, the above effect may suggest the active replication of PVB19 only in heart biopsies with ongoing inflammation due to MC or DCMi [33]. Patients with an inflammatory process in histopathological examinations of the heart specimen have PVB19 mRNA, unlike patients who are DNA-positive for PVB19, without ongoing inflammation [34].

Khatami A et al. confirm PVB19’s prevalence in 34.1% patients with DCM (96% CI: 23.8–46.1%). However, analyses suggest that the influence of PVB19 on inducing DCM was not statistically significant (OR: 1.163, 95% CI: 0.706–1.916; *p* < 0.553) [17].

The thesis of the influence of active PVB19 replication on DCMi is confirmed by clinical studies by Schultheiss HP and Bock CT et al. on the use of interferon-beta (IFN-β) to suppress active PVB19 transcription in the presence of DCMi. The supply of IFN-β over a period of 6 months led to a decrease in mean NT-proBNP values (548 ± 204 pg/mL vs. 319 ± 59 pg/mL, *p* = 0.001) and improved performance in the NYHA class, as shown in Table 2.

LVEF also improved significantly (51.6 ± 14.1% vs. 61.0 ± 17.5%, *p* = 0.03). In the placebo group, LVEF deteriorated in 80% of subjects from mean baseline values of 52.0 ± 20.0% to 42.0 ± 17.9% after 6 months. The normalization of the parameters by the elimination of active PVB19 replication suggests a clear impact of the virus on myocardial function and the risk of the further development of DCMi [35].

#### 3.2.2. Other Selected Viral Etiological Factors Responsible for the Induction of Myocarditis

##### Coxsackie A and B Viruses

Among the viruses of the enterovirus genus, Coxsackie B3 (CVB3) viruses were considered the species most frequently causing DCMi in the years 1975–1985. A small molecule of single-stranded viral RNA has mechanisms that enable infiltration into the body’s cardiomyocytes by binding to the CAR receptor with the participation of the DAF coreceptor [9,36]. The infiltration process begins with the first, acute phase, associated with intensive viral replication in cardiomyocytes along with the production of immunologically non-specific inflammatory mediators leading to damage to myocardial cells. In the second phase of infection, the host’s immune system turns on, causing an inflammatory reaction with autoimmune features. There are two main response mechanisms. Cross-reactions between the virus and cardiomyocyte epitopes and the exposure of intracellular structures of damaged cardiomyocytes. The development of numerous antibodies, including those against the heavy chains of alpha and beta myosin, impairs the contractility of cardiac muscle cells. This disturbs the ionic balance of cells, leading to overload with Ca^2+^ ions. Patients with developed viral MS also have antibodies against beta adrenergic receptors, M-2 muscarinic receptors or troponin. The third phase of inflammation is a multifactorial stage that can lead to both the improvement and deterioration of myocardial performance. The cessation of the inflammatory process, usually associated with the disappearance of the virus in cardiomyocytes, leads to improved cardiac systolic function and an increase in LVEF in 50–70% of cases. Chronic CVB3 infection, increased inflammation or excessive damage to the myocardium during the previous phases may lead to permanent systolic dysfunction of the heart chambers, a decrease in LVEF and subsequent post-inflammatory dilated cardiomyopathy [36,37].

##### Herpesvirus 6 (HHV6)

Herpesviruses subtypes 6A and 6B belong to the group of cardiotropic viruses. The mechanism of MC induction by HHV6 remains unclear. It is known that HHV6 uses CD4+ lymphocytes and endothelial cells with the CD46 receptor [9]. However, based on endomyocardial biopsy (EMB), it is suspected that this virus plays a significant role in inducing viral CM and subsequent DCMi. As in the case of PVB19, to determine that the etiological factor of viral CM is HHV6, it is necessary to confirm replication at the level of ˃500 copies of viral DNA in 1 microgram of myocardial DNA [4]. Moreover, HHV6 is considered one of the factors initiating the reactivation of latent PVB19 infection, leading to the previously described consequences [32,38].

##### Epstein–Barr Virus (EBV)

Epstein–Barr virus, which is the etiological factor of infectious mononucleosis and post-transplantation lymphoproliferative diseases, is responsible for acute MC in rare cases (˂1%). The mechanism of cardiomyocyte infection is the infection of B lymphocytes. This virus also has the ability to infiltrate surrounding tissues. Damage to myocardial tissue results from the initiation of inflammatory processes, the secondary autoimmune response and endothelial cell dysfunction [9].

##### Cytomegalovirus (HHV5/HCMV)

Cytomegalovirus has the ability to infect and enter the latent phase for a significant part of the host’s life. Cells of the epithelium, endothelium, smooth muscle and fibroblasts are the target point for the pathogen. It is estimated that, depending on the region, 30–90% of the population is infected with it. The transition from the latent phase to active infection is characteristic of immunocompetent patients. In this group of patients, the incidence of MC induced by HCMV ranges from 15 to 30%, with a significantly worse prognosis and higher mortality [9].

##### Hepatitis C Virus (HCV)

The hepatitis C virus has so far been associated mainly with liver damage, cryoglobulinemia, glomerulonephritis and other diseases not related to the heart muscle. However, its ability to accumulate immune complexes and activate and modulate the autoimmune response places it in the light of a potential cardiotropic pathogen. Additionally, this is supported by its significant global spread, covering approximately 58 million people. On average, 6.3% to 37% of patients with viral myocarditis or DCMi are HCV positive. The mechanism of inflammation induction is unknown. It is known that the target cells for HCV are monocytes and CD68+ macrophages, which influence the modulation of the immune response. Most likely, TNF-α, as one of the pro-inflammatory factors, initiates the inflammatory cascade causing the overproduction of nitric oxide and calcium imbalance, which ultimately leads to cardiomyocyte damage [9].

##### Influenza A and B (H1N1) Virus

In the general population, influenza A virus of the H1N1 subtype is considered one of the important etiological factors inducing viral MC. It is estimated that H1N1 infection is responsible for 11% of myocarditis incidents [39]. Type B influenza viruses are much less likely to cause MS, but they are associated with a much worse course and higher mortality compared to type A [40]. Cardiomyocytes are not target cells for the H1N1 virus, but the ability to stimulate the immune system and overexpress trypsin, matrix metalloproteinases, cytokines and TNF-α promotes post-influenza damage to cardiomyocytes and is one of the most dangerous, potentially fatal complications of improperly treated H1N1 infections [41]. Acute fulminant MC results in a rapid decline in cardiac hemodynamic performance, cardiogenic shock and potentially fatal complex arrhythmias. In the group of pediatric patients, it is responsible for 30–40% of MC incidents, with a mortality rate of up to 48% [39].

The above data are summarized briefly in Table 3.

### 3.3. Discussion

Nowadays, disorders of the physiological response of the immune system and the accompanying new autoimmune diseases are more common. This dramatically changes the role of viruses in inducing MC and the resulting heart failure. Modern imaging and histopathological and immunohistochemical techniques used in medicine allow for the more and more precise detection and identification of etiological factors of MC, even long after the incident. This made it possible to attribute the etiology to existing idiopathic cases of MC with ventricular dysfunction. Improved identification techniques have resulted in the dominant group A and B coxsackieviruses declining in importance in favor of parvovirus B19. The ability of PVB19 to infect early in human life and remain in the latent phase, in light of the increasing frequency of immunological disorders, favors the reactivation of infections. PVB19 infection is characterized by a very diverse clinical picture and prognosis, depending on the patient’s age, gender, genetic predisposition and environmental factors—from asymptomatic forms with slight damage to the ventricular function to an echocardiographic picture of dilated cardiomyopathy and symptoms of advanced heart failure. The small number of reports in this area and inaccuracies resulting from ongoing research and ongoing changes make it impossible to clearly answer the question of whether PVB19 is a factor inducing de novo MC and dilated cardiomyopathy or only accompanies the above conditions. However, clinical studies conducted on large cohorts suggest that PVB19 is a viral etiological agent capable of causing de novo MC along with dilated cardiomyopathy. Due to the small number of papers reporting on the mechanisms of the inflammatory induction of viral etiologic agents in causing myocarditis, it seems helpful to pay attention to animal models, which may also be accompanied by hyperlipidemia. Hyperlipidemia is a significant risk factor for acute cardiac events due to its association with oxidative stress. This leads to arterial wall remodeling, including an increase in the thickness of the intima media complex (IMT) and endothelial dysfunction leading to plaque formation. The decreased nitric oxide synthesis and accumulation of lipids in the wall result in a reduction in the vasodilating potential of the vessel. This study aimed to establish a clear relationship between markers of endothelial dysfunction and the activity of repair enzymes in cardiac tissue from a pig model of early atherosclerosis. The study analyzed markers of oxidative stress formed during lipid peroxidation processes, including etheno DNA adducts, ADMA and NEFA. These markers play a crucial role in reactive oxygen species analysis in ischemia–reperfusion and atherosclerosis in mammalian tissue. Essential genes involved in oxidative-stress-induced DNA demethylation like OGG1 (8-oxoguanine DNA glycosylase), MPG (N-Methylpurine DNA Glycosylase), TDG (Thymine-DNA glycosylase), APEX (apurinic/apirymidinic endodeoxyribonuclease 1), PTGS2 (prostaglandin-endoperoxide synthase 2) and ALOX (Arachidonate Lipoxygenase) were measured using the Real-Time RT-PCR method. The data suggest that high oxidative stress, as indicated by TBARS levels, is associated with high levels of DNA repair enzymes and depends on the expression of genes involved in the repair pathway. In all analyzed groups of heart tissue homogenates, the highest enzyme activity and gene expression values were observed for the OGG1 protein recognizing the modified 8oxoG. Conclusion: With the long-term use of an unbalanced diet, the levels of all DNA repair genes are increased, especially (significantly) Apex, Alox and Pigs, which strongly supports the hypothesis that an unbalanced diet induces oxidative stress that deregulates DNA repair mechanisms and may contribute to genome instability and tissue damage [42,43,44,45,46,47,48,49]. The insufficient number of reports prevents the establishment of clear guidelines for the management of PVB19-induced myocarditis. Therefore, it is necessary to conduct multicenter clinical trials taking into account all clinical aspects, as well as differences in the sensitivity and specificity of PVB19 detection depending on the techniques used, in order to provide answers in the future. Since there are not enough data on this topic in the professional and scientific literature, there is a need to explain their role, which is the main intention of this article.

## 4. Conclusions

In light of recent data, PVB19 should be considered as one of the main viral etiological agents of MC and DCM. Despite a small number of reports, significant effects of PVB19 infection on LVEF and NT-proBNP levels have been shown. In addition, these results confirm the effect of IFN-β treatment in patients with DCMi. IFN-β treatment leads to an improvement in LVEF and NYHA class performance with a decrease in NT-proBNP levels.

## Figures and Tables

**Table 1:** Correlation between the inflammation, ejection fraction and etiology of the disease. Inflammatory dilated cardiomyopathy (DCMi); non-inflammatory dilated cardiomyopathy (DCM); cardiac ejection fraction (EF) [31].

Correlation between the Inflammation, Ejection Fraction and Etiology of the Disease				
	DCMi EF > 45%	DCMi EF < 45%	DCM EF > 45%	DCM EF < 45%
Number of patients	816	282	1663	584
PVB19-negative	72.2	57.9	71.5	79.8
PVB19-positive	20.4	33.3	23.9	17.6
Other viral etiological factors	7.4	8.8	4.6	2.6

**Table 2:** New York Heart Association (NYHA); interferon-beta (IFN-β) [35].

Improvement in the NYHA Class in Patients Included in the Suppression of PVB19 Replication with the Use of IFN-β				
	NYHA I	NYHA II	NYHA III	NYHA IV
Initially [number of patients]	0	5	8	0
After 6 months of IFN-β therapy [number of patients]	7	5	1	0

**Table 3:** Summary of the incidence, clinical course and therapeutic management of myocarditis according to the viral etiologic agent. Parvovirus B19 (PVB19); Coxsackie B3 (CVB3); Herpesvirus 6 (HHV6); Epstein–Barr Virus (EBV); Cytomegalovirus (HCMV); Hepatitis C virus (HCV); Influenza A virus subtype (H1N1); Myocarditis (MC); Dilated cardiomyopathy (DCM); Left ventricle ejection fraction (LVEF); Tumor necrosis factor alfa (TNF-α); Interferon beta (IFN-β) [1,9,39].

Viruses	Incidence of Myocarditis	Clinical Course	Therapeutic Investigation
PVB19	56–73%	The virus penetrates the endothelial cells of myocardial blood vessels and progenitor cells of the erythroid line Inflammatory proteins cause abnormalities in the myocardial microcirculation with the subsequent lysis of cardiomyocytes and impaired cardiac performance Transition of infection into the chronic phase may result in DCM	Anti-inflammatory investigation (drugs for fever and mild pain). Immunosuppressive treatment (IFN-β)
CVB3	10–20%	Non-specific inflammatory reaction Cross-reactions between virus and cardiomyocyte epitopes with cardiomyocyte damage Regression of inflammation with improvement in cardiac performance or permanent systolic dysfunction, decreased LVEF and post-inflammatory DCM	Anti-inflammatory investigation (drugs for fever and mild pain). Immunosuppressive treatment and IV immunoglobulin. Antivirals treatment (pleconaril, interferon, and acyclovir)
HHV6	0.8%	Unclear mechanism of myocarditis infection Infection may induce the reactivation of the latent form of PVB19	No clear data available Symptomatic treatment depending on the clinical manifestation
EBV	<1%	Infection of B lymphocytes Damage to myocardial tissue with inflammatory and autoimmunological processes Lack of information about the clinical course due to the rare frequency of EBV-induced MC	No clear data available Symptomatic treatment depending on the clinical manifestation
HCMV	15–30%	Infection of the epithelium, endothelium, smooth muscle and fibroblasts Specific for immunocompetent patients Responsible for fatal cases of MC	No clear data available Symptomatic treatment depending on the clinical manifestation
HCV	6.3–37%	Unknown mechanism of inflamation induction Monocytes and CD68+ macrophages were target cells for HCV Pro-inflammatory factors initiate the inflammatory cascade, causing the overproduction of nitric oxide and calcium imbalance, leading to cardiomyocyte damage and cardiac dysfunction	Symptomatic treatment depending on the clinical manifestation Standard HCV antivirals treatment
H1N1	11%	Viruses stimulate the immune system with the overexpression of trypsin, matrix metalloproteinases, cytokines and TNF-α Post-influenza cardiomyocytes damage Acute fulminant MC with a rapid decline in the cardiac hemodynamic performance, cardiogenic shock and, potentially, fatal complex arrhythmias	Treatment based on hemodynamic and ventilatory support Other symptomatic treatment depending on the clinical manifestation

## Data Availability

On request of those interested.
